# Supplementary material for: Deletion of wfs1 Impairs Oligodendrocyte Precursor Cells Dorsal Distribution and Myelination Through the wfs1-hmgcs1 Axis in Zebrafish
Source: Biology (Basel). 2026 Aug 16;15(16):1402. doi: 10.3390/biology15161402 (PMC13510151; doi:10.3390/biology15161402)
Supplement: Supplementary file 1 [file biology-15-01402-s001.zip › File S1-Transcriptome sequencing data and differentially expressed genes (DEGs) in wfs1-- zebrafish at 5 dpf/2.KEGG/wfs1KOvsWT/DOWN/src/dre04814.html]

dre04814


- dre:556023

- Down regulated genes

556023(-1.34487366325401)

- dre:474327

- Down regulated genes

474327\_1(-5.01402477912525)

- dre:336681

- Down regulated genes

406811\_1(-1.09337424046752)

- dre:334274

- Down regulated genes

334274(-1.53299620220276)
- dre:100149148

- Down regulated genes

100149148(-1.048436973731)

- dre:100037358

- Down regulated genes

100037358\_1(-1.14225112918381)

- dre:337226

- Down regulated genes

337226(-2.98693262894109)

- dre:30429

- Down regulated genes

30429\_2(-1.78714384880949)

- dre:57934

- Down regulated genes

57934(-1.25539807424297)

- dre:353247

- Down regulated genes

353247(-1.16019504230133)

- dre:353247

- Down regulated genes

353247(-1.16019504230133)

Close
